# Nonlinear Optical Studies of Gold Nanoparticle Films

**DOI:** 10.3390/nano9020291

**Published:** 2019-02-19

**Authors:** Anuradha Rout, Ganjaboy S. Boltaev, Rashid A. Ganeev, Yue Fu, Sandeep Kumar Maurya, Vyacheslav V. Kim, Konda Srinivasa Rao, Chunlei Guo

**Affiliations:** 1 The Guo China-US Photonics Laboratory, State Key Laboratory of Applied Optics, Changchun Institute of Optics, Fine Mechanics and Physics, Chinese Academy of Sciences, Changchun 130033, China; anuradharout@ciomp.ac.cn (A.R.); ganjaboy_boltaev@mail.ru (G.S.B.); 15541103381@163.com (Y.F.); sandeep@ciomp.ac.cn (S.K.M.); mik750594@rambler.ru (V.V.K.); ksrao@ciomp.ac.cn (K.S.R.); 2 The Institute of Optics, University of Rochester, Rochester, NY 14627, USA

**Keywords:** gold thin film, nonlinear absorption, nonlinear refraction, transient absorption, nanoparticles, high-order harmonics

## Abstract

Gold films are widely used for different applications. We present the results of third- and high-order nonlinear optical studies of the thin films fabricated from Au nanoparticle solutions by spin-coating methods. These nanoparticles were synthesized by laser ablation of bulk gold in pure water using 200 ps, 800 nm pulses. The highest values of the nonlinear absorption coefficient (9 × 10^−6^ cm W^−1^), nonlinear refractive index (3 × 10^−11^ cm^2^ W^−1^), and saturation intensity (1.3 × 10^10^ W cm^−2^) were achieved using 35 fs, 400 nm pulses. We also determined the relaxation time constants for transient absorption (220 fs and 1.6 ps) at 400 nm. The high-order harmonic generation was studied during propagation of 35 fs, 800 nm pulses through the plasma during the ablation of gold nanoparticle film and bulk gold. The highest harmonic cutoff (29th order) was observed in the plasma containing gold nanoparticles.

## 1. Introduction

The quantum confinement effect allows the distinguishing of the parameters of nanoparticles with regard to the bulk materials. Among the metals suited for nanoparticle preparation for optoelectronics and nonlinear optics, one can distinguish silver [1,2], copper [3,4], and gold [5]. The further search for prospective materials in nanoparticle formation, their preparation, and application are of considerable importance. Meantime, the unique properties of low-dimensional materials have ignited numerous studies of their characteristics [6,7,8].

An interest in nanoparticle-containing films is developing due to their enhanced nonlinear optical response [9]. Such films have attracted interest due to their potential applications in optoelectronics as optical switches and optical limiters. The development of new thin film compounds containing both semiconductor and metal nanoparticles (NPs) allows for further enhancement of the nonlinear optical characteristics of such structures. During the last decade, there has been an interest in the nonlinear optical features of chalcogenide thin films [10,11,12,13]. The investigations of these films have shown their prospects as optical limiters.

Most of previous nonlinear optical studies of different materials in a strong electromagnetic field were performed using solutions and thick (of the order of a few micrometers) films. At the same time, it is interesting to investigate thin (of order of a hundred nanometers) films. Particularly, gold nanocomposites have tremendous applications in various fields due to the influence of their surface plasmon resonance (SPR) on the optical properties [14]. In the past decade, researchers have demonstrated the potential applications of gold nanoparticles (NPs) using different lasers [15,16]. In those studies, the NPs were produced by laser ablation of the gold bulk targets. Laser ablation is an efficient method for the synthesis of NPs using different pulse durations, pulse energies, as well as various liquids [17]. In most cases, the deionized water was used during the ablation to produce the gold NPs. Several studies have revealed variable nonlinear optical properties of gold NPs, nanorods, and thin films using different laser pulses [18,19,20,21,22]. Particularly, the optical nonlinearities of Au NP arrays, which were determined by performing Z-scan measurements using a femtosecond laser (800 nm, 50 fs), were reported in [21]. The third-order nonlinear optical properties of gold NPs embedded in Al_2_O_3_, ZnO, and SiO_2_ at the wavelength of 532 nm using the nanosecond Nd:YAG laser were analyzed in [22]. Previously, the high-order harmonics from gold NPs were studied in [23]. One can assume that the plasma produced on the thin films can enhance the efficiency of high-order harmonics due to the influence of NPs.

In this paper, we analyze the third-order nonlinear optical properties and transient absorption of gold NP thin film as well as demonstrate the high-order harmonic generation (HHG) from the ablated gold NP thin film.

## 2. Experimental Arrangements

**Figure 1:**
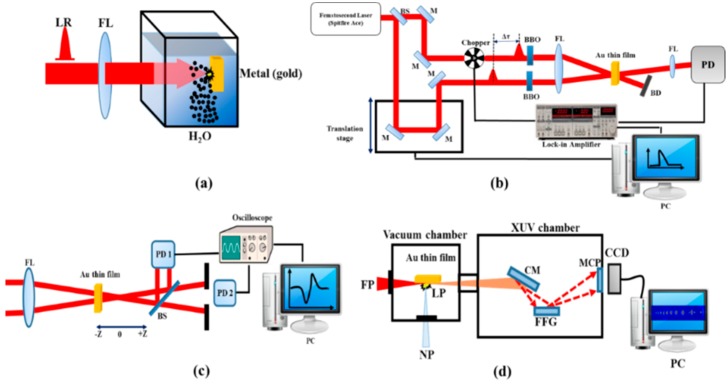
(**a**) Schematic of laser ablation. LR, laser radiation; FL, focal lens. (**b**) Schematic of the transient absorption measurements. BS, beam splitter; M, mirrors; FL, focal lenses; PD, photo diode; BD, beam dumper; BBO, barium borate crystal; PC, personal computer; (**c**) Schematic of Z-scan. FL, focusing lens; PD1, photodiode 1; PD2, photodiode 2. (**d**) High-order harmonic generation (HHG) setup. FP, converting femtosecond pulses; NP, heating nanosecond pulses; LP, laser plasma; CM, cylindrical gold-coated mirror; FFG, flat field grating; MCP, microchannel plate; CCD, charge-coupled device camera.

Figure 1a shows the scheme for the synthesis of Au NPs in solution by irradiation of the bulk gold target immersed in deionized water using 800 nm, 200 ps, 1 kHz pulses. The laser beam was focused by a 100 mm focal length lens on the bulk gold. Typically, the energy density of laser radiation on the metal surface was in the order of 10 J/cm^2^. The irradiation of metal surface resulted in the fast removal of the material confined to the laser spot. The sample was displaced with regard to the laser beam, using a translating stage to avoid the formation of deep holes. The ablation was done for 10 min at continuous stirring. The Au film was prepared by evaporation of the aqueous suspension containing gold nanoparticles and then used as the target to perform the experiments. The thickness of thin film was examined by scanning electron microscope (SEM) (HITACHI S-4800, Tokyo, Japan) to be approximately 100 nm.

A noncollinear degenerate pump–probe technique was employed to measure the transient absorption (TA) in Au NP films (Figure 1b). A TA study was performed using the 400 nm radiation obtained by second harmonic generation of 800 nm, 35 fs pulses using a 2 mm thick barium borate (BBO) crystal. Prior to conversion of 800 nm to 400 nm, the laser pulse was split into two pulses using a 30:70 beamsplitter. Pump and probe pulses were focused to the sample using a 300 mm focal length lens. The transmitted probe radiation was detected by an ultrafast Si photodiode (DET025AL, Thorlabs, China) connected to a lock-in-amplifier to measure the transmittance of the probe pulse with respect to the position of the motorized stage. The lock-in-amplifier was externally triggered by the optical chopper running at 300 Hz.

Figure 1c shows the Z-scan scheme, which was used for the third-order nonlinear optical studies in Au NP thin films. The laser radiation (800 nm, 30 fs, 1 kHz) was focused on the sample by a 40 cm focal length lens. The thin film was placed near to the focus of the beam. The sample was scanned along the beam direction by a computer-controlled translation stage. After passing through the sample the transmitted beam was then split by a 50:50 beamsplitter. The closed aperture (CA) and open aperture (OA) Z-scans at 30 nJ probe energy were used to characterize the nonlinear absorption and refraction of the Au NP thin film.

The HHG in the plasmas produced during the ablation of Au NP thin film was performed using the setup shown in Figure 1d. The driving femtosecond pulses (800 nm, 30 fs, 1 kHz) propagated through the plasma formed by the nanosecond heating pulses (1064 nm, 5 ns, 10 Hz) at different delays between the heating and driving pulses. The harmonic yield was maximized by adjusting the position of the target. The generated high-order harmonics were analyzed by an extreme ultraviolet (XUV) spectrometer and detected by a microchannel plate with phosphor screen. The harmonic spectrum from the phosphor screen was imaged by a charge-coupled device (CCD) camera.

## 3. Results and Discussion

### 3.1. Low-order Nonlinearities of Au NP Film

**Figure 2:**
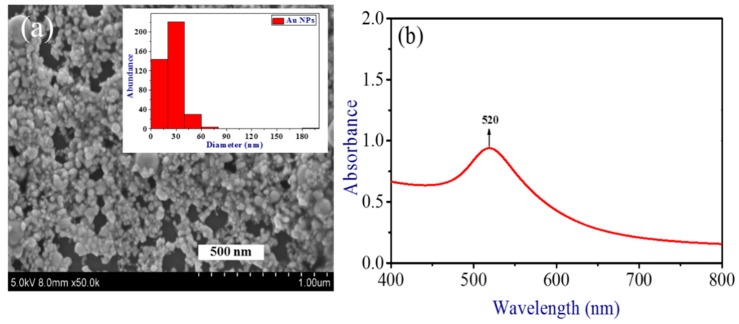
(**a**) SEM image and size distribution of Au NPs. (**b**) Absorption spectrum of Au NP thin film.

SEM images and histograms of the Au NPs prepared by ablation of bulk gold using picosecond pulses are presented in Figure 2a. The inset in Figure 2a shows the size distribution of Au NPs, which covers the 10–90 nm range with mean size 30 nm. The sample shown in Figure 2a was prepared by drying the drop of Au NP solution on the Si wafer or glass. Then this sample was analyzed by SEM. A few existing empty places in the SEM image had the sizes (<200 nm) significantly smaller than the area used for absorption measurements (5 × 5 mm^2^) of the thin (~100 nm) gold film deposited on the silica glass plate. The presence of those tiny holes causes the insignificant variation of a whole spectral pattern. The absorption spectrum remained the same in different parts of deposited film due to the averaging of absorbance measured along the large area.

The absorption spectrum of thin film was analyzed in the range of 400 to 800 nm. The absorbance measurements were made using a 100 nm thin film. Figure 2b shows the absorption spectrum of Au NP thin film. The observed surface plasmon resonance of Au NPs was at 520 nm.

The Z-scan has proven to be a most versatile technique for the measurements of the lowest-order optical nonlinearities of different materials. Two schemes (OA and CA) are frequently applied to measure the nonlinear absorptive and refractive properties of matter. The use of these two configurations is a prerequisite for the accurate measurements of nonlinear refractive index (*γ*) and nonlinear absorption coefficient (*β*). Though the latter parameter can also be retrieved using the CA scheme, the accuracy of those measurements is lower compared with the OA scheme. The beam width of the probe radiation in the focal plane was 76 µm (full width at half maximum), i.e., a thousand times larger than the sizes of nanoparticles (30 nm). Correspondingly, the nonlinear optical response was accumulated from the large amount of particles presented inside the focal spot area. What we see in the SEM hardly can be classified as the aggregates. Actually, they are the separated nanoparticles placed close to each other. The thickness of the film (100 nm) assumes that only a few (actually 3 to 4) NPs comprise the whole active path of this film. Thus, the response occurs from a large amount of separate Au NPs acting as the nonlinear refractive/absorptive material.

The Z-scan of the normalized transmittance caused by the influence of saturable absorption (SA) and reverse saturable absorption (RSA) is described as [24,25]:

(1) $$T_{SA,RSA}\left(z\right)=\left(1-\frac{q}{2\sqrt{2}}\right)\times \left(1+\frac{I_{0}}{I_{sat}\;\left(1+x^{2}\right)}\right).$$

Here *q* = *βI*_0_*L*_eff_/(1 + *z*^2^/*z*_0_^2^), *x = z/z*_0_, *z_o_* = *k*(*w*_0_)^2^/2 is the Rayleigh length, *k* = 2*p*/*λ* is the wave number, *I*_0_ is the peak intensity in the focal plane, *L*_eff_ *=* (1–exp (–*α*_0_*L*))/*α*_0_ is the effective length of the medium, *w*_0_ is the beam waist radius at the 1/e^2^ level of intensity distribution, *α*_0_ is the linear absorption coefficient, *L* is the thickness of sample, and *I*_sat_ is the saturated intensity of the medium.

In the case of the CA Z-scan, the normalized transmittance of nonlinear refraction and absorption (NRA) is given as [26]:

(2) $$T_{NRA}\left(z\right)=1+\frac{2\left(-\rho x^{2}+2x-3\rho \right)}{\left(x^{2}+1\right)\left(x^{2}+9\right)}\Delta \emptyset_{o}.$$

**Figure 3:**
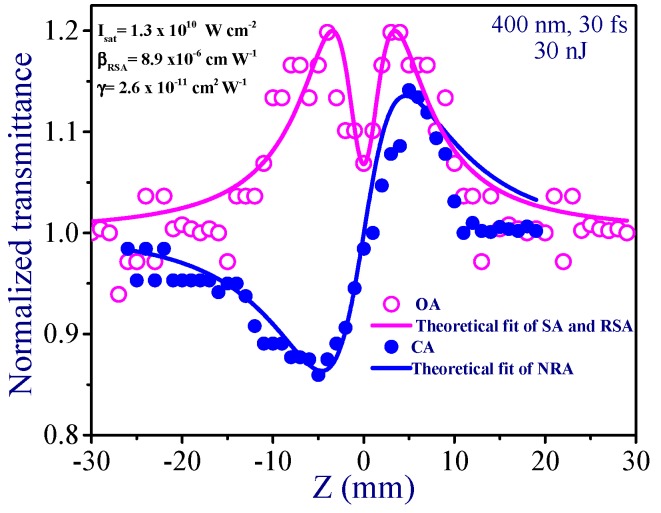
Open aperture (OA) and closed aperture (CA) Z-scan curves of thin film measured using the 400 nm, 30 fs pulses. SA is saturable absorption; RSA is reverse saturable absorption; NRA is nonlinear refraction and absorption.

Here *ρ* = *β*/2*kγ* and Δ*Φ*_0_ = *kγI*_0_*L*_eff_ is the phase change due to nonlinear refraction. The investigation of the nonlinear optical characteristics of Au NP thin film was carried out using the CA and OA Z-scans under excitation by 400 nm, 30 fs probe pulses at the same pulse energy (30 nJ). Figure 3 shows the OA and CA Z-scan curves of thin film. In the case of OA, the Au NP thin film showed SA, while close to focal area it demonstrated the RSA.

The fitting of the curve comprising the influence of saturable absorption and reverse saturable absorption was accomplished using Equation (1), similarly to the technique described in [24,25]. This equation contains the saturation intensity (*I*_sat_), which was determined during the fitting procedure, alongside the nonlinear absorption coefficient of reverse saturable absorption. The measured saturated intensity was 1.3 × 10^10^ W cm^−2^. *β*_RSA_ was calculated from the fitting curve to be 9 × 10^−6^ cm W^−1^. This value is one of largest reported in the case of thin films measurements using different materials.

In the case of CA measurements, the self-focusing was observed. The nonlinear refractive index was calculated from the fitting of the Equation (2) with the experimental CA curve. The fitting allowed for the determination of two parameters (Δ*Ф*_0_ = *kγI*_0_*L*_eff_ and *ρ* = *β*/2*kγ*) required for definition of the nonlinear refractive index and nonlinear absorption coefficient. Correspondingly, by knowing Δ*Φ*_0_, *k*, *I*_0_, and *L*_eff_ one can easily determine *γ*. The nonlinear refractive index was calculated to be 2.6 × 10^−11^ cm^2^ W^−1^.

**Figure 4:**
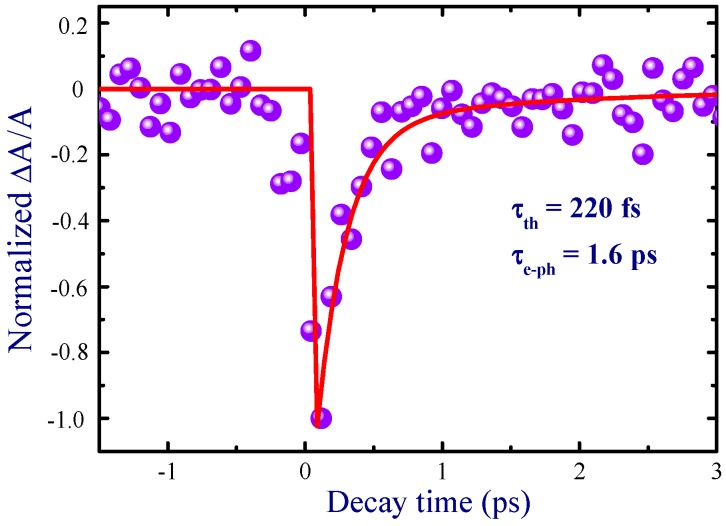
Pump–probe dynamics of Au NP thin film at 400 nm.

Time evolution of the absorption of probe pulses in the presence of pump pulses in the Au NP film at 400 nm is shown in Figure 4. Prior to the TA measurements of the Au NP film deposited on a glass slide, the TA measurements of the pure glass slide were performed to separate its contribution from the former TA data. The pump–probe profile for thin gold film indicated the process of photobleaching due to excitation of the NPs under irradiation of 52 nJ, 400 nm, 35 fs pump pulses. In general, mechanisms of photoexcitation by femtosecond pulses in a metal include the excitation of electrons to a higher energy state through optical absorption, which leads the nonequilibrium electronic subsystem to relax via redistribution of the energy of excited electrons, typically known as electron–electron scattering interaction. The energy redistribution of the excited electrons occurs through electron–electron scattering within about 100–300 fs, which have been attributed to the electron thermalization process [27,28]. Another process of relaxation for excited electrons occurs via energy transfer of the electron to the lattice in the picosecond time scale due to electron–phonon interaction. In the present study, the employed pulse width (35 fs) of the femtosecond pulses was smaller than the decay time scale of the electron–electron relaxation dynamics in Au NPs. This made it feasible to probe the electron–electron dynamics. The following phenomenological response function was used to determine the time constants associated with electron–electron and electron–phonon interactions [28]:

(3) $$f\left(t\right)=H\left(t\right)\left(1-exp\;\left(-t/\tau_{\mathrm{th}}\right)\right)\;\left(exp\;\left(–t/\tau_{e-ph}\right)\right)$$

Here $\tau_{th}$ and $\tau_{e-ph}$ are electron thermalization time constant and electron–phonon relaxation time constant, respectively, and H(t) is the Heaviside step function, which is equal to 0 if t<0 and 1 if t>0. The fitting of the TA profile (Figure 4, solid line) allowed determination of these time constants ($\tau_{th}$ = 220 fs and = 1.6 ps) at *λ* = 400 nm.

The relaxation time constant *τ_th_* was associated with the electron thermalization process in Au NPs [29]. There are several reports on the study of electron thermalization kinetics, which validate the above-mentioned approach [30,31,32,33].

### 3.2. High-order Harmonic Generation in Au NP Plasmas

The nanostructured materials can significantly enhance the harmonic yield in XUV range, since they have already demonstrated the ability to increase the second- and third-order nonlinear optical processes [23]. Below we report the studies of high-order nonlinear optical processes in the plasma produced on the Au NP thin film deposited on glass substrates. We compared the HHG from the plasmas created on the bulk gold target and Au thin film. The harmonics generated from the plasmas produced on the glass substrates, without thin film, were negligible compared with those from the plasmas produced on the bulk and metal thin films. In two cases, the plasma plumes were ablated using the 1064 nm, 5 ns heating pulses. The moderate intensity of heating pulses allowed the evaporation of the neutral atoms and singly-charged ions from the targets during laser ablation. The advantages of the applications of neutral atoms and singly-charged ions for HHG have been demonstrated in a series of previous HHG studies in the laser-produced plasmas [34,35,36]. The optimal plasma plume was created by moving the target with regard to femtosecond beam propagation.

**Figure 5:**
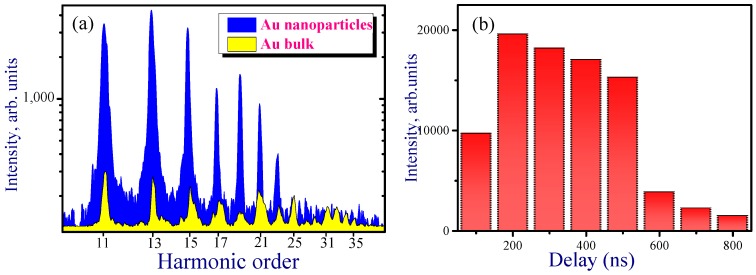
(**a**) High-order harmonic spectra generated in the plasmas produced on the Au thin film and bulk Au. (**b**) Dependence of 11th harmonic yield on the delay between heating and driving pulses.

Figure 5a shows the harmonic spectra from the plasmas produced on the bulk Au and Au NP-contained thin films at 200 ns delay time between the heating and driving pulses. It was demonstrated that the harmonic intensity was significantly (approximately five times) enhanced in the case of Au NP thin film with regard to bulk Au. The experiments were carried out at different delays between the heating nanosecond and driving femtosecond pulses. Figure 5b shows the intensity variation of the eleventh harmonic with respect to different delay times. The maximum intensity of harmonics was achieved in the range of 200–500 ns delay times. During the ablation of thin film at optimal delays, the generation of the strong harmonics up to the 29th order was achieved. However, after crossing the delay of 800 ns the yield of harmonics was notably decreased.

The HHG from gas clusters has been reported in a few earlier studies [37,38,39,40,41]. Moreover, the harmonic generation in the plasmas containing plasmonic particles of different materials has also been analyzed to demonstrate the advantages of the proposed approach of HHG amendment [42,43,44]. In the plasma HHG studies, the nanoparticles were synthesized during laser ablation in a vacuum prior to formation of the NP-containing plasma plume or their appearance in the plasma was accomplished by ablation of commercially available nanoparticles.

The present research has demonstrated the preparation of gold nanoparticles during ablation of solid material in the liquid environment, deposition of synthesized NPs on the glass and silicon wafer, and ablation of thin deposited film in a vacuum to produce the plasma containing nanoparticles of narrow size distribution for harmonic generation at different delays between heating and driving pulses. Among them the most important novelty is the analysis of the role of the delay between the heating picosecond pulses creating nanoplasma and the driving femtosecond pulses generating harmonics on the HHG conversion efficiency. All previous HHG studies with nanoparticle-containing plasmas and gases were carried out at either fixed delay between heating and driving pulses (in the former case) or fixed distance from the nozzle of the gas jet. In previous plasma HHG studies, short delays of up to 100 ns between the heating and driving pulses were employed. It was not clear how heavy nanoparticle species could influence the processes of frequency conversion, because there was no proof of their presence in the interaction region with the driving laser (i.e., at the distance of a few hundred micrometers from the target surface), since one can expect their arrival in the region of the femtosecond laser beam propagation a few tens of microsecond from the beginning of ablation. One explanation was based on the disintegration of larger species into small clusters and monoatomic species, which probably could reach the interaction area at the short delays employed. However, no sufficient confirmation of this assumption has been provided.

To match the propagation of the driving pulse and the highest concentration of the studied group of multi-atomic species with a much larger delay, which cannot be achieved by optical methods, one therefore has to use the electronic methods of the adjustment of the delay between the heating and the driving pulses. The application of two electronically separated pulses from different lasers synchronized by a digital delay generator allows analysis of the involvement of various multi-particle species in the HHG process. Application of this approach for HHG in multi-particle plasmas, alongside with other methods of harmonic enhancement, requires the analysis of the dynamics of ablated species spreading out from the target to temporally match them with the propagation of driving femtosecond pulses through the plasma. One can expect the arrival of 30 nm particles in the region of the femtosecond laser beam propagation a few tens of microseconds from the beginning of ablation. Meanwhile, our studies demonstrated the optimization of delays leading to the growth of harmonic yield in the case of Au NP plasma at around 200 ns. Below, we address this difference in the expected and actual optimal delay between heating and driving pulses in the case of Au NP-contained plasma.

In the case of a thermalized ablation plume, the average arrival times can be assigned to different cluster sizes. The delay between heating and driving pulses at which the harmonic yield reaches its maximum should scale as a square root of the atomic or molecular weight of the constituents. The ejection of lighter clusters from NPs allows them to reach the region of the driving beam earlier than heavier species. Therefore, NPs comprised of *n* atoms should appear in the interaction zone *n*^0.5^ times later compared to single atoms, molecules, or ions of the sulfides. Our additional studies revealed that, for bulk gold ablation, the maximum harmonic yield from single gold atoms and ions occurred at a delay of about 180–300 ns. Meantime, the Au NPs allowed efficient generation at about 200–400 ns delay (Figure 5b), which is approximately equal to the delay in the case of Au atoms and ions. Furthermore, attempts to observe HHG at the delays of up to 50 μs (i.e., at the expected delay for thermalized larger nanoparticles) did not show any harmonic emission. Thus, our studies demonstrated that NPs arrive at the area of interaction with the femtosecond laser beam notably earlier than one would expect for a thermalized ablation plume. In other words, all gold NPs acquire, from the very beginning, a similar kinetic energy and spread out from the surface with velocities approximately similar to those of the single gold atoms and ions ablating from bulk material. This conclusion reconciles the similarity in the optimal delays for HHG from bulk and NPs targets of the same material (Au).

It was suggested in [45] during their studies of third harmonic generation in plasma plumes that, at laser ablation characterized by the blast mechanism of laser–matter interaction, a similar average kinetic energy *E* = mv^2^/2 could characterize all plasma components of the same elemental composition. Thus, the average arrival time assigned to the particles containing different amounts of identical atoms will be approximately the same, contrary to the case of slowly produced thermalized plasma. Our studies of the high-order nonlinear optical processes occurring in the plasmas confirm this assumption. The difference in “optimal” delays between heating and driving pulses is related to the difference in the velocities of particles, which depends on the atomic masses of the components of NPs. Similar conclusions have been reached during recent studies of HHG from the metal sulfide quantum dots [46].

## 4. Conclusions

We have prepared gold nanoparticle-contained thin film by evaporation of the Au NP suspension, which was synthesized by laser ablation. The Au NP thin film was characterized using the UV-visible absorption spectra and SEM analysis. We have demonstrated the strong nonlinear absorption (9 × 10^−6^ cm W^−1^) in this film at 400 nm. The thin film exhibited a switch from SA to RSA at stronger excitation. The relaxation time of SA was measured to be 1.6 ps. The high-order harmonic generation was analyzed in the plasma containing gold nanoparticles. The HHG efficiency in that case was ten times higher compared with the case of bulk gold ablation. For the first time, the effective application of the ablated 100 nm thin film of gold nanoparticles for harmonic generation in the 27–115 nm range was achieved.
